# Delineation of traditional village boundaries: The case of Haishangqiao village in the Yiluo River Basin, China

**DOI:** 10.1371/journal.pone.0279042

**Published:** 2022-12-15

**Authors:** Lixuan Liu, Zijian Liu

**Affiliations:** 1 School of Art and Design, Shaanxi University of Science and Technology, Xi’an, China; 2 School of Architecture, North China University of Water Resources and Electric Power, Zhengzhou, China; Institute for Advanced Sustainability Studies, GERMANY

## Abstract

In the conservation and development of traditional villages, it is vital to delineate reasonable village boundaries. At present, China’s traditional village boundaries are mainly defined through the boundaries of village construction land in its land policy. This approach ignores the "natural" state of traditional village boundaries and does not truly reflect villagers’ use of their villages. Using the constituent line segments of traditional village boundaries as parameters, we delineate a series of traditional village boundaries and constructed an assessment system for traditional village boundaries in terms of the convenience of villagers’ activities and their habitual use of village space. We used the delineation of the boundary of Haishangqiao village in the Yiluo River Basin in China as an example to test the effectiveness of the traditional village boundary delineation method and assessment system. The results show the following: (1) The virtual line segments connecting the buildings on the traditional village boundary are the main factors affecting the delineation of the traditional village boundary. (2) The amplitude of vibration and the global deviation of traditional village boundaries are the main influencing factors of complexity. (3) The complexity of traditional village boundaries is negatively correlated with the convenience of villagers’ activities. (4) The complexity of traditional village boundaries is negatively correlated with the emptiness of the internal space of traditional villages. The results of this study can provide practical reference values for scholars of rural planning, rural construction, rural conservation, rural management, and rural research.

## Introduction

According to the definition given in the relevant documents of the Ministry of Housing and Urban–Rural Development of China and another four national ministries, traditional villages refer to “villages that were formed earlier, owns rich traditional resources, is relatively complete at present and has high historical, cultural, scientific, artistic, social and economic values” [1]. Mr Feng Gicai said, "Traditional villages contain a wealth of historical information and cultural landscapes, and are the greatest legacy of China’s agrarian civilisation" [2]. With the rapid urbanization process, traditional villages are generally facing problems such as physical aging, functional decline, and constructive destruction [3, 4]. In particular, when selected as a traditional village, the State allocates special funds for the repair and development of the village, accelerating the financial impact on the fragile built environment and especially on the rich natural systems [5]. Therefore, the conservation and sustainable development of traditional villages have become important issues in the field of rural planning and construction [5, 6]. A large part of the work of village planning and construction revolves around the elements of village buildings, streets, and public facilities. It is these elements that constitute the characteristic spatial form of a village, especially for traditional villages. Therefore, the conservation and sustainable development of traditional villages are inseparable from spatial forms.

The spatial pattern of traditional villages includes architectural space, street space, and overall space [7]. The formation of traditional villages is a natural growth process. The spatial pattern of traditional villages is usually gradually generated by self-organization without any interference of planning activities and is a form of bottom-up organization [8–10]. For example, if the site selection principle of each house in a village is to avoid arable land and flooding hazards, and be close to the foothills, then the village will have a circular or semi-circular layout if it is in hilly areas, and a strip layout if it is in valleys [11]. The internal man-made environment and the external natural environment are organically integrated and interdependent. As described in the Ministry of Housing and Urban–Rural Development’s Traditional Villages Evaluation and Recognition Index System (for Trial Implementation): “The village maintains a harmonious and symbiotic relationship with the surrounding beautiful natural landscape environment or traditional rural scenery”[12]. The necessary construction actions required for the conservation and sustainable development of traditional villages should be conducted within traditional villages to reduce their impact on the external natural environment and to protect the overall space of said traditional villages. It is therefore important to delineate the boundaries between the man-made built environment inside traditional villages and the external natural environment.

"Boundaries" have been the focus of research in many disciplines. Linguistic [13], cultural [14], cadastral parce [15], cultural heritage [16], urban growth [17, 18], and administrative boundaries are all virtual boundaries that have been artificially defined. The natural boundaries formed by mountains, rivers, fences, and the Great Wall of China are all physical boundaries [19, 20]. However, the traditional village boundary, which is of great concern in urban and rural planning and architecture, is the line of demarcation between a rural settlement and the external natural environment. Except in some ancient rural settlements, the boundary exists in the form of a physical wall, moats, etc. [21]. In most traditional villages, it is a combination of physical boundaries (the edges of buildings) and non-physical boundaries (the gaps between buildings) [22]. As non-physical boundaries can be delineated in a variety of ways, this makes traditional village boundaries complex, ambiguous, and uncertain, which in turn affords them a rich diversity of forms [23]. Therefore, the reasonable delineation of traditional village boundaries is the primary prerequisite for the conservation and sustainable development of villages [19, 24, 25].

The delineation of traditional village boundaries is widely used in urban and rural development. Rapid urbanization has also led to dramatic changes in rural settlements globally, and the use of remote sensing data to quantify changes in rural settlement boundaries can reveal phenomena such as the disappearance, shrinkage, expansion, and merging of villages [26]. Urban growth boundaries (UGBs) have been adopted globally as a policy tool to control urban sprawl, with village administrative boundaries used in UGB delineation to adjust delineation outcomes [18]. In the process of tourism development in ancient villages, interviews and observations are used to explore how the underlying boundaries are created and developed and what their impacts are, contributing to boundary studies by examining the local boundary formation process [27]. Tao [28] studied the external spatial interface of a settlement, and Meng et al. [29] analyzed the landscape morphology of a rural settlement boundary at the landscape level.

A methodology for the delineation of traditional village boundaries is based on satellite remote sensing data. Lionel et al. proposed a method for extracting village boundaries from very high-resolution optical satellite imagery that can be used for regional- and national-scale areas with minimal human effort [30]. Ding and Wang used SPOT-5 imagery as a key spatial analysis data source, and rural settlements were classified into three types: Valley settlements, slope settlements, and mountain settlements, according to the different geographical locations in which they are located, and their remote sensing interpretation was used to delineate boundaries in response to their boundary characteristics [31].

Another methodology for the delineation of traditional village boundaries is based on field research data. Chamara J. et al. used PGIS interviews and a group discussion method with PRM steps to explore participatory geographic information system (PGIS) techniques for delineating land boundaries along the eastern boundary of Wilpattu National Park in Sri Lanka [24]. Beccy and David compared three methods—circular buffers, unweighted Voronoi polygons (sometimes referred to as Thiessen polygons), and multiple-weighted Voronoi polygons—to estimate the boundaries between villages in the West African agricultural landscape [25]. Oliskiewicz-Krzywicka described the origins of village boundary shapes that were formed in the Middle Ages and presented the rules on which the delineation was based [32].

Another methodology for the delineation of traditional village boundaries is based on a closed curve finding algorithm. Pu [33] used three different imaginary boundary scales (7, 30, and 100 m) to produce planar closure graphs of rural settlement boundaries. Lu [34] chose the boundary scales of 12, 30, and 200 m and refined the programming algorithm, positing that the scale of 12 m, which is equivalent to twice the depth of a standard village building, is more appropriate. Dong [35] used a network diagram of building nodes to find the convex hull between two buildings at a specific influence distance and superimposed them to find the boundary contour of a settlement. Zhang [36] applied the Delaunay triangular network principle to visualize the spatial relationships between buildings, and combined the minimum spanning tree algorithm and the convex hull principle to extract the cluster boundaries.

Despite this large body of literature pertaining to quantification methods for traditional village boundaries, these studies have mainly delineated traditional village boundaries from a macro-perspective, while there is a lack of research on the delineation of traditional village boundaries from a micro-perspective. Moreover, very little literature can be found on assessing the merit of delineating boundaries. In this paper, the physical and virtual boundaries of traditional villages were traversed and extracted from a micro-perspective. Based on the convenience of village use and the habitual use of space by villagers, boundary delineation was evaluated for its superiority, and the boundary with the best convenience and habitual use was selected as the final traditional village boundary result to guide the construction, protection, and sustainable development of traditional villages.

Our research questions were as follows: (1) There is a certain ambiguity and uncertainty in the delineation of traditional village boundaries, so what factors influence the outcome of boundary delineation? We attempted to analyze and discuss the attributes of traditional village boundaries in terms of their constituent elements. (2) How to assess the superiority of a traditional village boundary and what indicators are used and how to quantify them? (3) What are the relationships between the assessment indicators? What are the characteristics of the indicators involved in the assessment and how do they relate to each other, and which indicators have a significant impact on the assessment results? In response to these three questions, we propose to adopt a scientific and quantitative approach to boundary delineation, boundary assessment, and the relationship between assessment indicators from the perspective of traditional village users.

**Fig 1:**
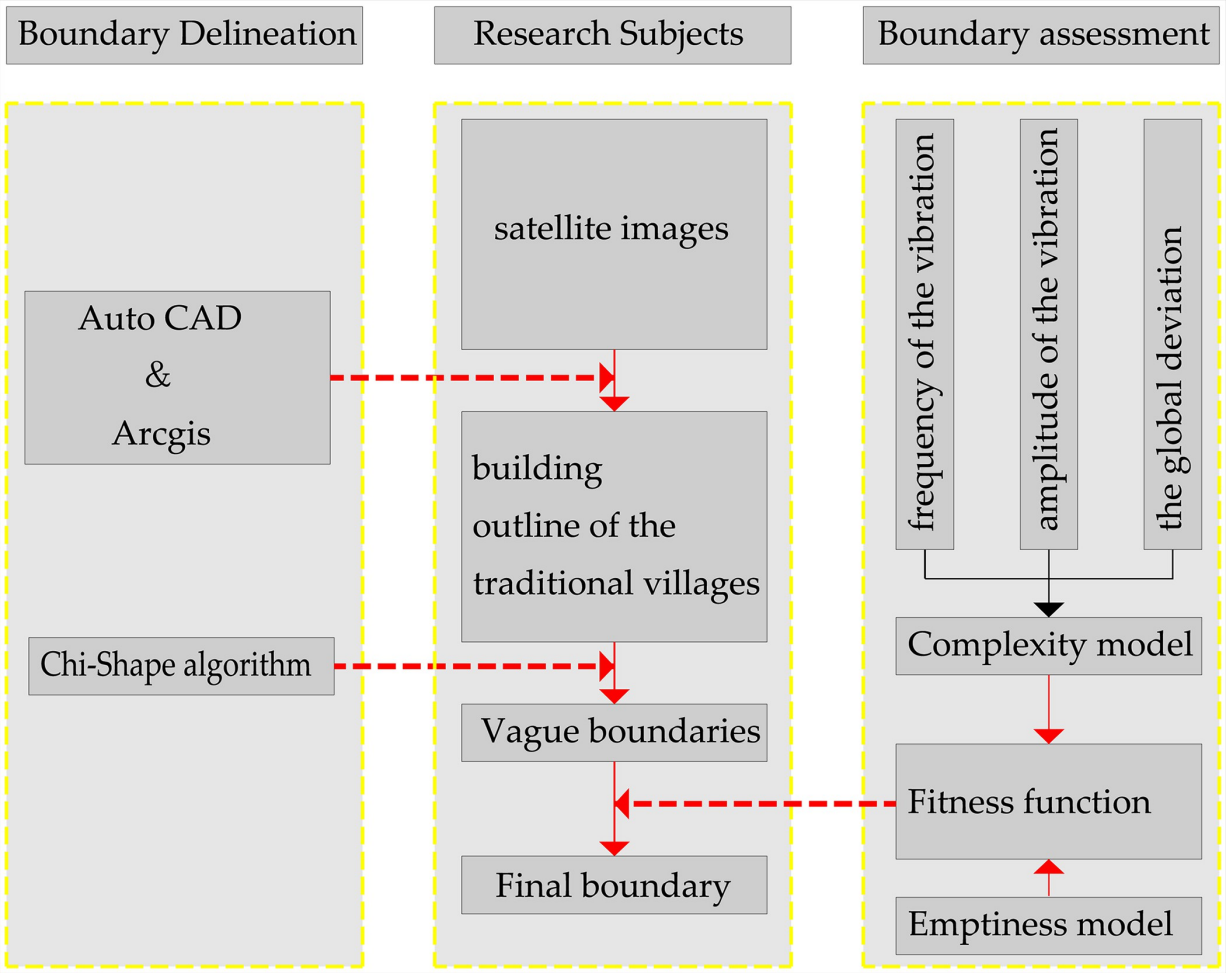
Workflow of research on the delineation of the boundary of Haishangqiao village.

The remainder of this paper is structured as follows: In Section 2, we introduce the study area and describe the data and data-preprocessing steps; in Section 3, we present the methodology and results; in Section 4, we discuss the results; and in Section 5, we draw conclusions. The workflow of this study is shown in (Fig 1).

## Study area and data description

**Fig 2:**
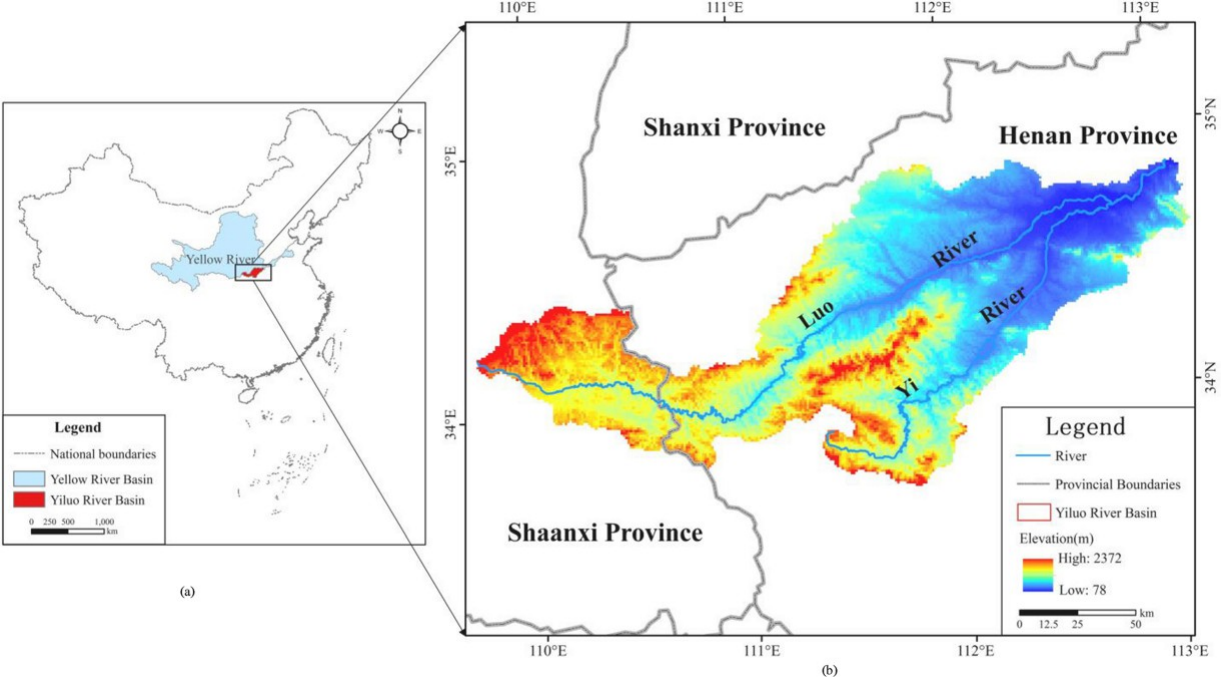
(a) Locations of the Yellow River Basin and the Yiluo River Basin; (b) watershed of the Yiluo River Basin. (This figure was cited from Fig 2 in article “Analysis and Prediction of Ecosystem Service Values Based on Land Use/Cover Change in the Yiluo River Basin” (https://doi.org/10.3390/su13116432)).

As shown in (Fig 2A), the Yellow River Basin is located in the central part of China [37]. Originating in the Bayan Har Mountains in Qinghai Province of western China, the Yellow River Basin empties into the Gulf of Bohai Sea in Shandong Province. The Yellow River Basin has an east–west extent of approximately 1900 km and a north–south extent of approximately 1100 km. Its total drainage area is around 795,000 km^2^. The terrain of the Yellow River Basin fluctuates greatly, and the elevation of the basin gradually decreases from west to east [37]. As shown in (Fig 2B), the Yiluo River Basin is a significant tributary in the Yellow River, covering an area of around 18,462.96 km^2^, including more than 20 counties in Shaanxi and Henan provinces [38]. The Yiluo River has two principal tributaries: The Luo River and the Yi River, where the Luo River is located on the north side and the Yi River on the south side [38]. The Yiluo River region is regarded as a core area of Chinese civilization in the Yellow River valley [39]. Therefore, the traditional villages in this region of China are highly representative.

Traditional villages, also called ancient villages, are nonrenewable resources that preserve cultural heritage and have substantial historical, cultural, scientific, social, esthetic, economic, and touristic value for local and international communities [40, 41]. In December 2012, the Ministry of Housing and Urban–Rural Development, the Ministry of Culture, and the Ministry of Finance of the People’s Republic of China declared their intention to publish a list of traditional Chinese villages and outlined the selection criteria for these villages [42]. Haishangqiao village (113°4´E-34°45´N) is an administrative village in Dayugou Town, Gongyi city, Henan Province, China, with 16 village groups, 561 households, 2302 people, 2865 mu of arable land, and an area of approximately 6.7 km^2^, as shown in (Fig 2B). On 6 June 2019, it was included in the fifth batch of Chinese traditional villages.

We derived the building outline data for Haishangqiao village by a satellite image. Firstly, we obtained the satellite image of Haishangqiao village from Landsat Science (http://landsat.visibleearth.nasa.gov). Then, we used CAD and ArcGIS software through the man-machine interaction method to get the result data. The data for the village comprised the outline plan of all buildings, the spatial relationships of the buildings, and the geographic coordinates of the buildings. We extracted the data for Haishangqiao village based on the natural agglomerated clustering state of the settlement, which may slightly deviate from the administrative division of the village.

## Methodology

### Ethics statement

The field studies were conducted in Haishangqiao village, Dayugou Town, Gongyi City, Henan Province, China. The boundary of the village was taken as the study area. The local government and the masses supported this study.

### Chi-shape algorithm

Duckham et al. presented a simple, flexible, and efficient algorithm for constructing a possibly non-convex, simple polygon [43]. The algorithm is based on Delaunay triangulation of the input point set. The shape produced by the algorithm is controlled by a single normalized parameter [43], which we call chi. Each chi corresponds to a length of L. The value of L in this study was determined as follows: First, we traversed all of the distances between the buildings on the outside of the village to find the maximum and minimum distances. Then, we divided the difference between the two distances into equal parts, and the number of parts depends on the accuracy required for L. In this paper, the difference in distance was divided into 100 equal parts. Finally, we added the value of each share to the minimum distance to form a column of distance values for L. The execution flow was as follows:

Generate the Delaunay triangulation of the point set P;

Delete the longest outside edge from these triangles according to the following principle: The deleted edge should be longer than the parameter L, and the outside edge of the triangle should still form a complete outline;

Repeat step 2 and continue to remove more edges;

Return one polygon at a time.

### Complexity model

Two factors are decisive for the intuitive rating of the complexity of a spatial object: The global shape of the object and the (local) vibration of its boundary [44]. Two measures are distinguished for the vibration: Its frequency and its amplitude. In order to describe the global shape of an object, the deviation of the object from its convex hull is additionally introduced [44]. The model contains three parameters—ampl, freq, and conv—and is formally defined as follows:

compl(P)=0.8*ampl(P)*freq(P)+0.2*conv(P) (2)

where ampl(P) is the vibration amplitude of the polygonal vertices, expressed as:

$$ampl(P)=\frac{boundary(P)-boundary(convexhull(P))}{boundary(convexhull(P))}$$ (3)

and freq(P) is the vibration frequency of the polygonal vertices, expressed as:

$$freq(P)=16*{\left({notches}_{norm}(P)-0.5\right)}^{4}-8*{\left({notches}_{norm}(P)-0.5\right)}^{2}+1$$ (4)

$${notches}_{norm}(P)=\frac{notches(pol)}{vertices(pol)-3}$$ (5)

and conv (P) is the convexity of the polygon, expressed as:

$$conv(P)=\frac{area(convexhull(p))-area(P)}{area(convexhull(p))}$$ (6)

where Boundary (P) is the length of the polygon, i.e., the perimeter of the traditional village boundary; Convexhull (P) is the convex hull of point set D, i.e., the convex hull of the traditional village; Area (P) is the area of the polygon, i.e., the area of the traditional village boundary; Notches (P) is the number of concave points in the polygon, i.e., the number of concave points in the traditional village boundary; Vertices (P) is the number of vertices in the polygon, i.e., the number of vertices in the traditional village boundary.

### Emptiness model

Akdag et al. developed the emptiness model [45], which assesses the emptiness of a polygon (P) with respect to a point set (D), based on Delaunay triangulation, as follows:

$$Emptiness(P,D)≔\frac{\left(\sum_{t\in DT{\left(D\right)}^{area(t)}>\theta^{inside(t,p)}}(area(t)-\theta )\right)}{area(P_{CONV})}$$ (7)

where P_conv_ is the convex hull of point set D, i.e., the convex hull of all the building outlines in a traditional village; DT (D) is the Delaunay triangle network of point set D, i.e., the Delaunay triangle network of the point set of the building outlines; θ is the threshold value for determining whether a triangle is empty, i.e., the average area of the Delaunay triangle network of point set D; t is a triangle whose area is potentially smaller than the threshold θ.

In Eqs (2)–(7), P represents the measured concave polygon and D represents the measured point set, i.e., P is the traditional village boundary and D is the point set of the building outlines.

### Fitness function

To generate a rural settlement boundary that neither contains a large empty area nor is too circuitous, Akdag et al. [45] proposed a fitness function that balances these two objectives. They defined the problem of fitting a polygon P to a set of spatial objects as follows:

ϕ(P,D)=Emptiness(P,D)+C*Complexity(P,D) (8)

where P represents the boundary of the rural settlement; D represents the set of points comprising the building outline; C is a parameter in the range [0, 1] that balances the emptiness and complexity of the boundary. The larger the value of C, the smoother the boundary. We set C to 1 to find smooth boundaries.

## Results

### Delineating the vague boundaries of Haishangqiao village using the chi-shape algorithm

**Fig 3:**
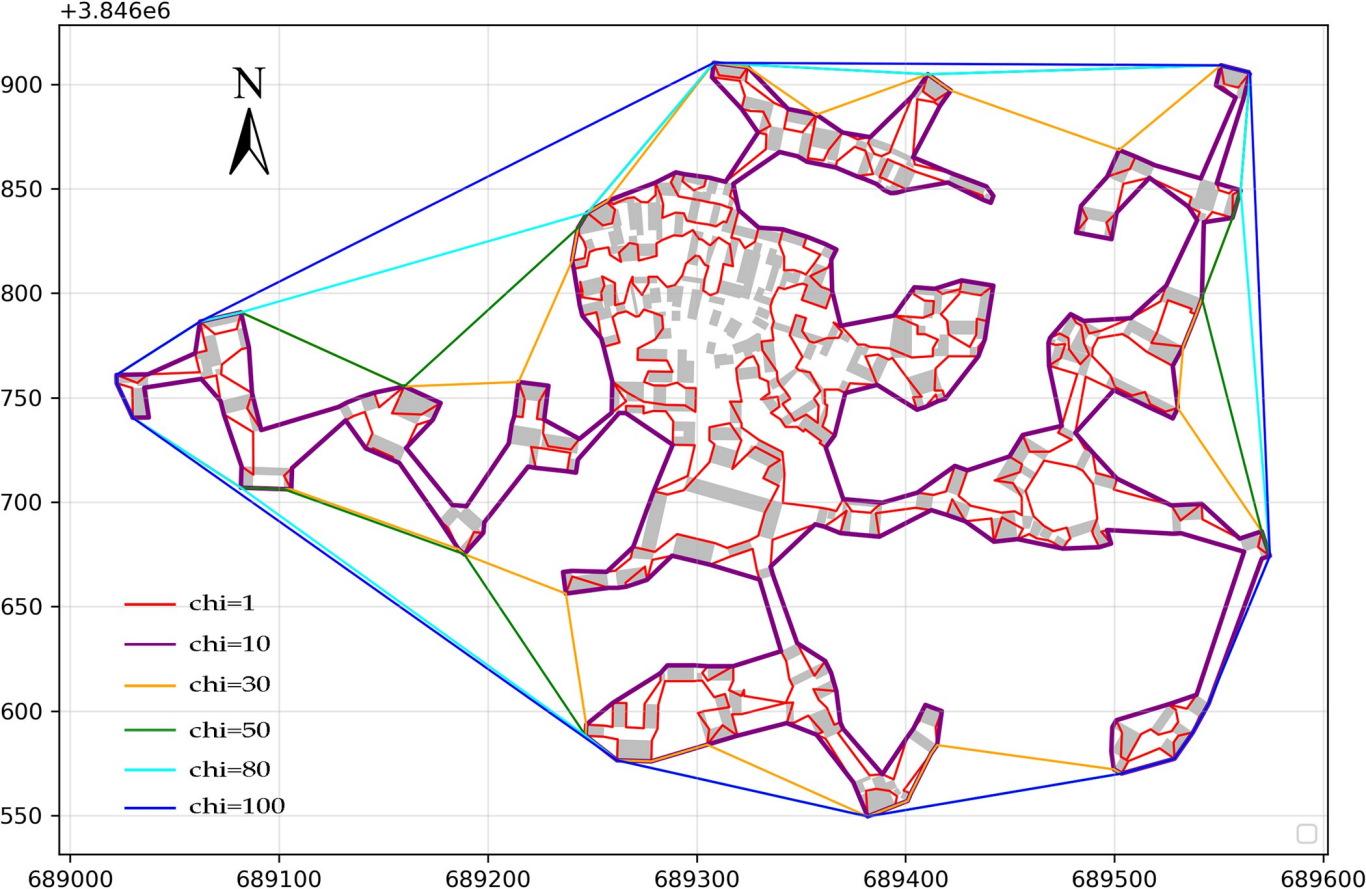
Part of the boundary of Haishangqiao village (when chi = 1, 10, 30, 50, 80, or 100) (The building outline data in Fig 3 was derived from Landsat Science (http://landsat.visibleearth.nasa.gov. This building outline is similar but not identical to the original image).

(Fig 3) shows the village boundaries generated for Haishangqiao village for different length parameters (chi). We can see from the figure that when chi = 1, the village boundary has many folds and the internal space is cramped, which cannot meet the normal use needs of villagers and cannot be called a village space in the true sense. When chi = 10, the village boundary has fewer folds and the spatial scale is moderate, which is in line with the characteristics of a village boundary. When chi = 30, 50, 80, or 100, the folding points of the village boundaries gradually decrease, but the internal space of the village also gradually loses its original compactness and becomes hollow, so that the internal buildings of the village lose their proper scale of an enclosure and cannot reflect the proper concentration of a village space.

### Evaluating the complexity of the vague boundaries of Haishangqiao village using the complexity model

A village is a gathering place where villagers live, and the village boundary is the outermost edge of villagers’ activities that is too convoluted to meet the requirements of ease of life. In order to judge the degree of complexity of a village boundary, we quantified the complexity of a village boundary by looking at three parameters: The amplitude of vibration, the frequency of vibration, and the global deviation of the village boundary curve. The lower the complexity, the greater the convenience at the village boundary and the more convenient it is for village use.

#### Assessment of the frequency of the vibration of the boundaries of Haishangqiao village

**Fig 4:**
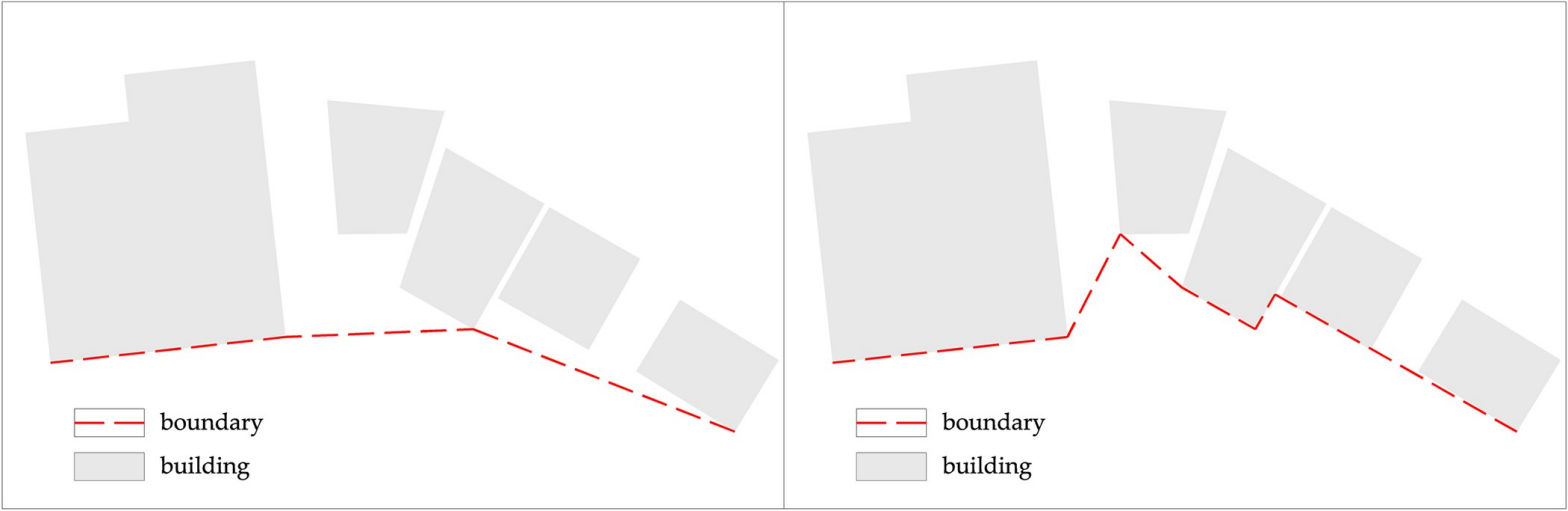
An illustration of the turn of direction of the village boundary.

**Fig 5:**
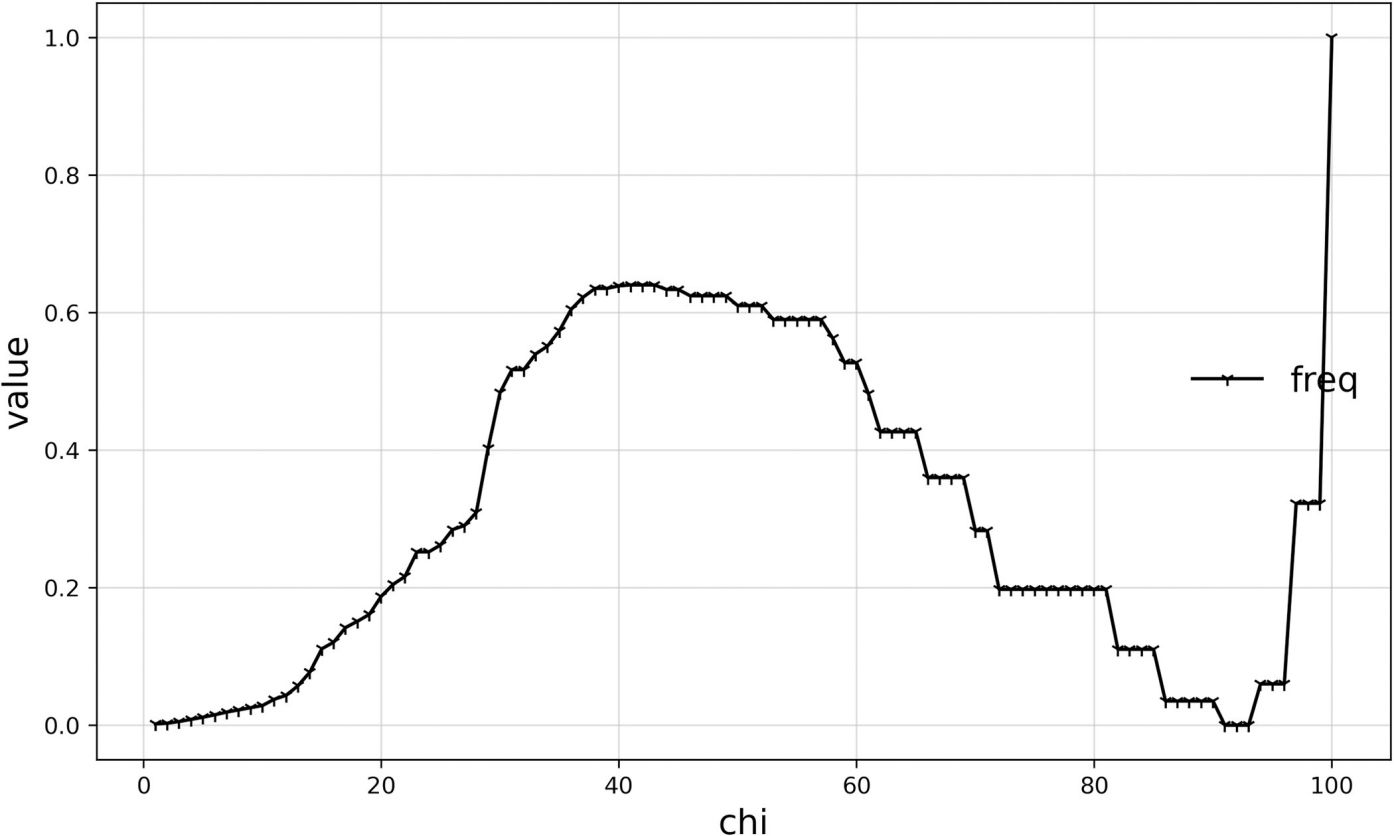
Curve of frequency of the vibration of Haishangqiao village’s boundaries.

The vibration frequency of the village boundary is the number of times the direction of the boundary fold switches, reflecting the frequency with which the direction of the village boundary changes. That is, whether two buildings connected by a virtual line segment are in a straight line. If they are not in a straight line, the direction has changed once. As shown in (Fig 4) the direction of the boundary changed two times in (Fig 4A) and five times in (Fig 4B). (Fig 5) shows the frequency of Haishangqiao village’s boundary vibration as a function of chi. We can see from the results that the frequency of vibration at the village boundary increases and then decreases with the value of chi, reaching a maximum at chi values of 41, 42, 43. When 0 < chi < 41 (42, 43), the vibration frequency increases with the chi value; when 43 < chi <91 (92, 93), the village boundary vibration frequency decreases with an increase in chi; when 93 < chi ≤100, the dynamic frequency increases again with the chi value. It indicates that as the length of the virtual line segment increases, the number of boundary direction changes also increases until the virtual line segment length reaches 117.12–122.78 m, when the direction change frequency starts to decrease. At the point where the length of the virtual line segment reaches 258.57–264.23 m, the frequency of directional transitions drops to a minimum.

#### Assessment of the amplitude of the vibration of the boundaries of Haishangqiao village

**Fig 6:**
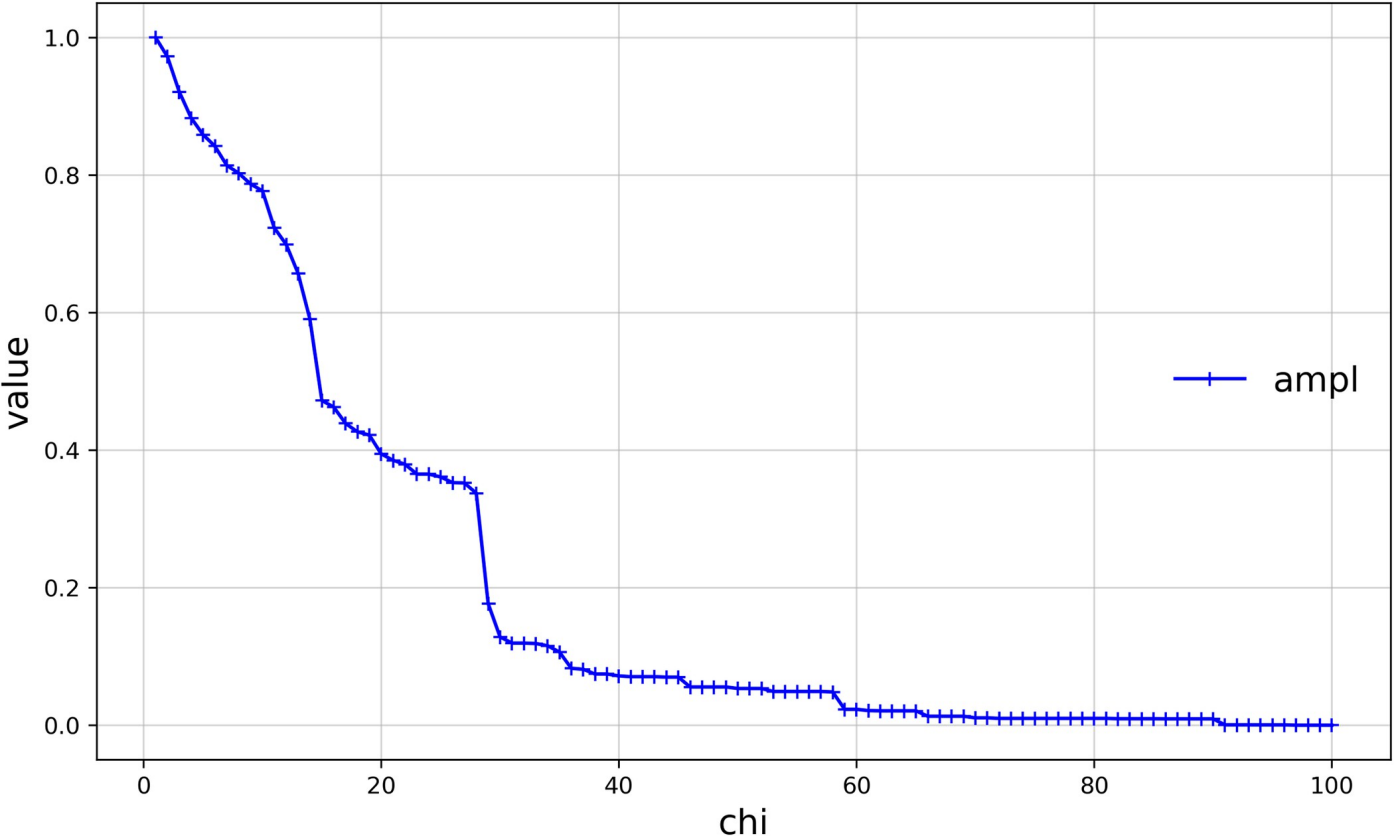
Curve of the amplitude of the vibration of the boundaries of Haishangqiao village.

The amplitude of vibration at the village boundary is the degree of depression between the notches formed by the turning of the boundary and reflects the choice of the location of the folding point of the line connecting the gaps between the buildings at the village boundary. As shown in (Fig 4), the degree of depression in (Fig 4A) is less than that in (Fig 4B). We used the ampl model to calculate the amplitude of vibration at the village boundary. (Fig 6) shows the vibration amplitude of Haishangqiao village’s boundaries as a function of chi. We can see from the results that the vibration amplitude of the village boundary is inversely proportional to chi. The vibration amplitude of the boundary decreases as chi increases, with a clear inflection point at chi = 14, 28, and 29. When 0 < chi < 14, the slope of the curve is steep and changes rapidly; when 14 < chi < 28, the slope of the curve becomes smaller and decreases more slowly; when 28 < chi < 29, the slope of the curve becomes larger again and decreases rapidly; after that, the chi curvature flattens out and there are no more obvious abrupt changes. This is because, after chi = 14 and 29, the decrease in the perimeter of the boundary becomes weaker and the value of the vibration amplitude decreases slowly, resulting in a gentle curve; at chi = 28, the decrease in the perimeter of the boundary increases and the value of the vibration amplitude decreases rapidly, resulting in a steep curve.

#### Assessment of the global deviation of Haishangqiao village’s boundaries

**Fig 7:**
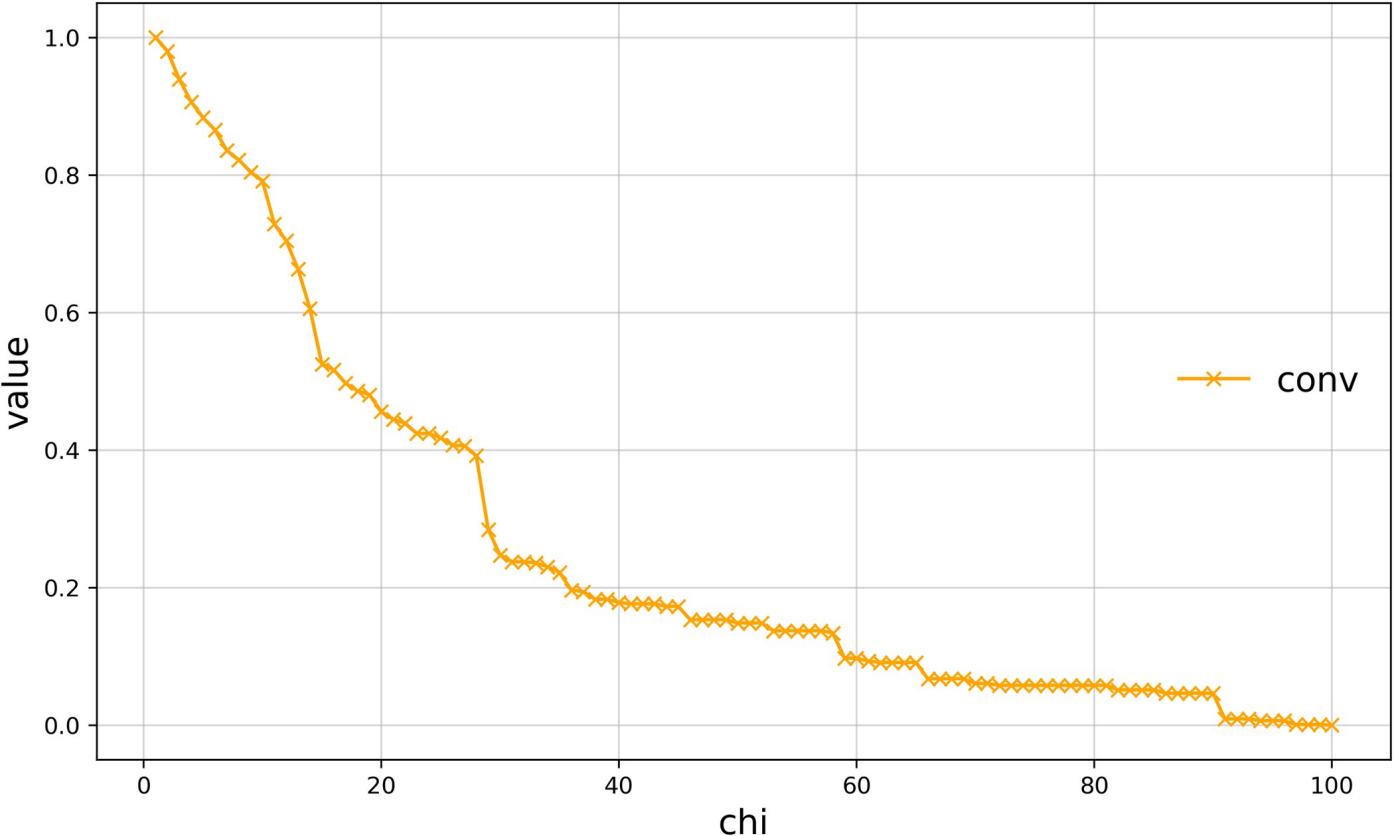
Curve of the global deviation of Haishangqiao village’s boundaries.

The global deviation of a village boundary is the degree of deviation of the boundary from its convex envelope and reflects the degree of global nature of the village boundary. It is the deviation of the village boundary from its smoothest curve. We used the con algorithm to calculate the global deviation of the village boundaries. (Fig 7) shows the global deviation of Haishangqiao village’s boundaries as a function of chi. We can see from the results that the global deviation of the village boundaries is inversely proportional to chi. The trend of the overall deviation curve is consistent with the trend of the vibration amplitude of the boundary. The overall value is slightly higher than the amplitude of the vibration of the boundary.

#### Complexity of the boundaries of Haishangqiao village

**Fig 8:**
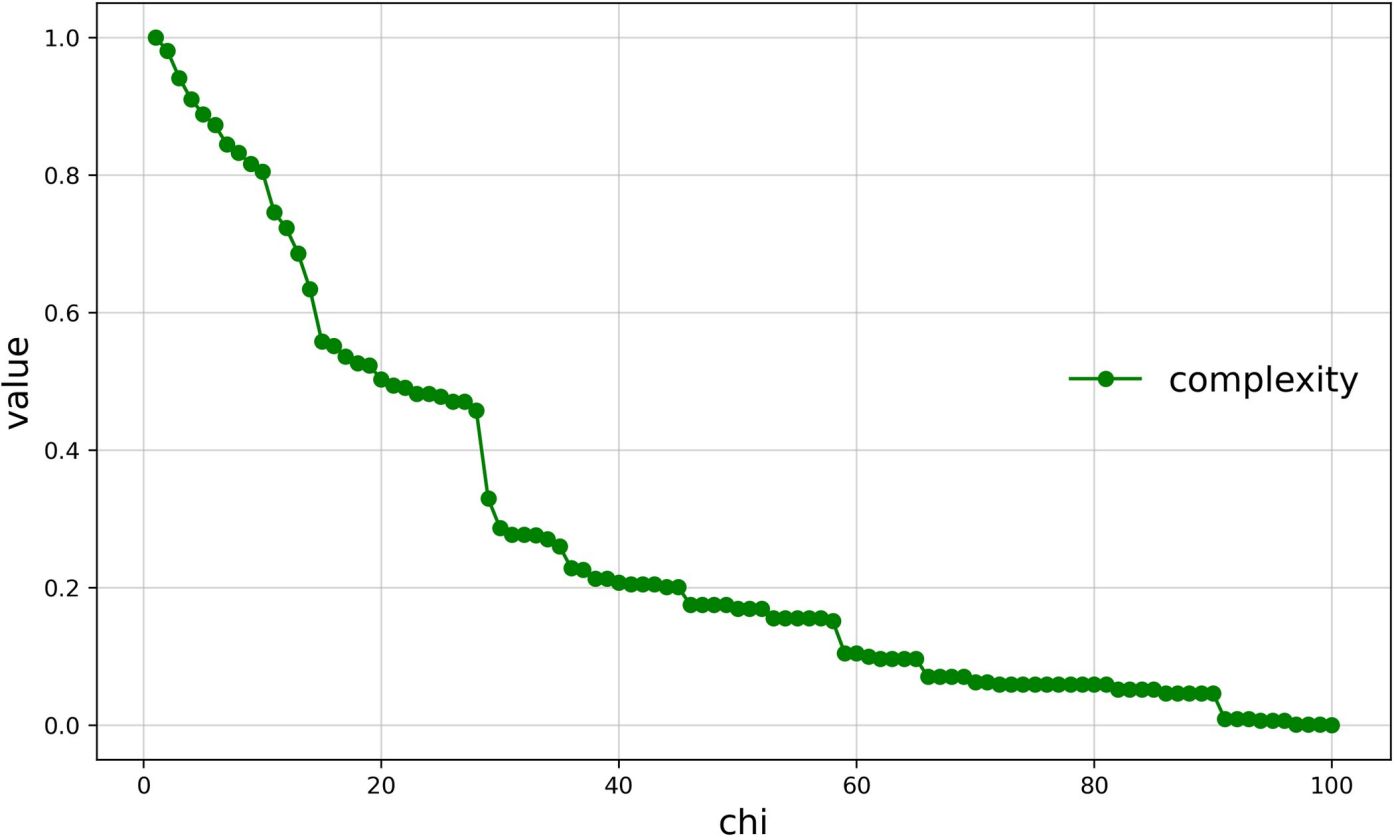
Curve of complexity of Haishangqiao village’s boundaries.

We combined the three parameters into a single value of complexity for description in order to make the boundary complexity description clearer. (Fig 8) shows the relationship between Haishangqiao village’s boundary complexity and chi. We can see that the complexity of the village boundaries is inversely proportional to chi. The trend of the complexity curve of the boundaries is consistent with the amplitude of the vibrations of the inflection point and the boundaries and the global deviation of the boundary. It shows that the amplitude of vibration and deviation of the village boundaries are the main factors affecting the complexity of said boundaries, and the higher the amplitude of vibration of the boundaries, the higher the complexity.

### Evaluating the emptiness of the boundaries of Haishangqiao village using the emptiness model

The external space at village boundaries is a public activity space within a village; it should not be too empty and should have a certain enclosure requirement. Therefore, spaces that are too large and lack enclosure are not considered as village public activity spaces and cannot be included within village boundaries. How do we determine the area of public space that is in line with villagers’ habits? We divided the village space into a number of triangular areas and took the average area of the village space as the threshold; those larger than the average area were considered as public spaces that do not conform to the custom and should be excluded from the scope of the village boundaries. We used the emptiness indicator to quantify the emptiness within each boundary. A smaller emptiness inside a boundary means that the space formed inside the boundary is more compatible with the villagers’ usage needs.

**Fig 9:**
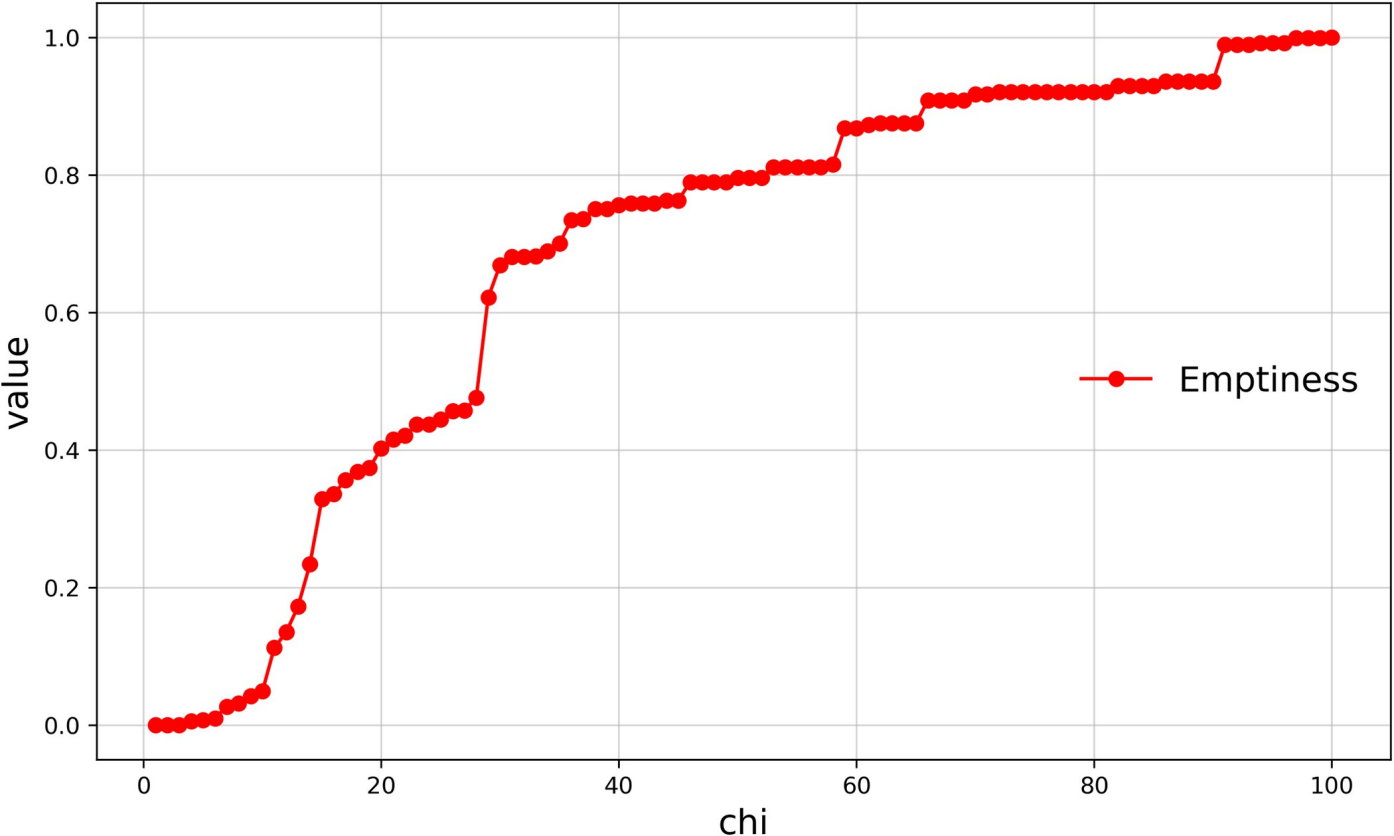
Curve of emptiness of Haishangqiao village’s boundaries.

Emptiness refers to the degree of emptiness of the space within village boundaries and is an indicator of the area properties of a polygon, reflecting the compactness of the internal space formed by the virtual line segments enclosing the buildings. (Fig 9) shows the relationship between the emptiness of Haishangqiao village’s boundaries and chi. We can see that the emptiness of the village boundaries is proportional to chi. The emptiness of the boundary increases as chi increases, with a clear inflection point at chi = 14, 28, and 29. The steep increase in the area of enclosed space within the village boundaries at these three chi values can be seen in (Fig 9), resulting in a clear inflection point.

### Delineating the boundaries of Haishangqiao village using the fitness function

**Fig 10:**
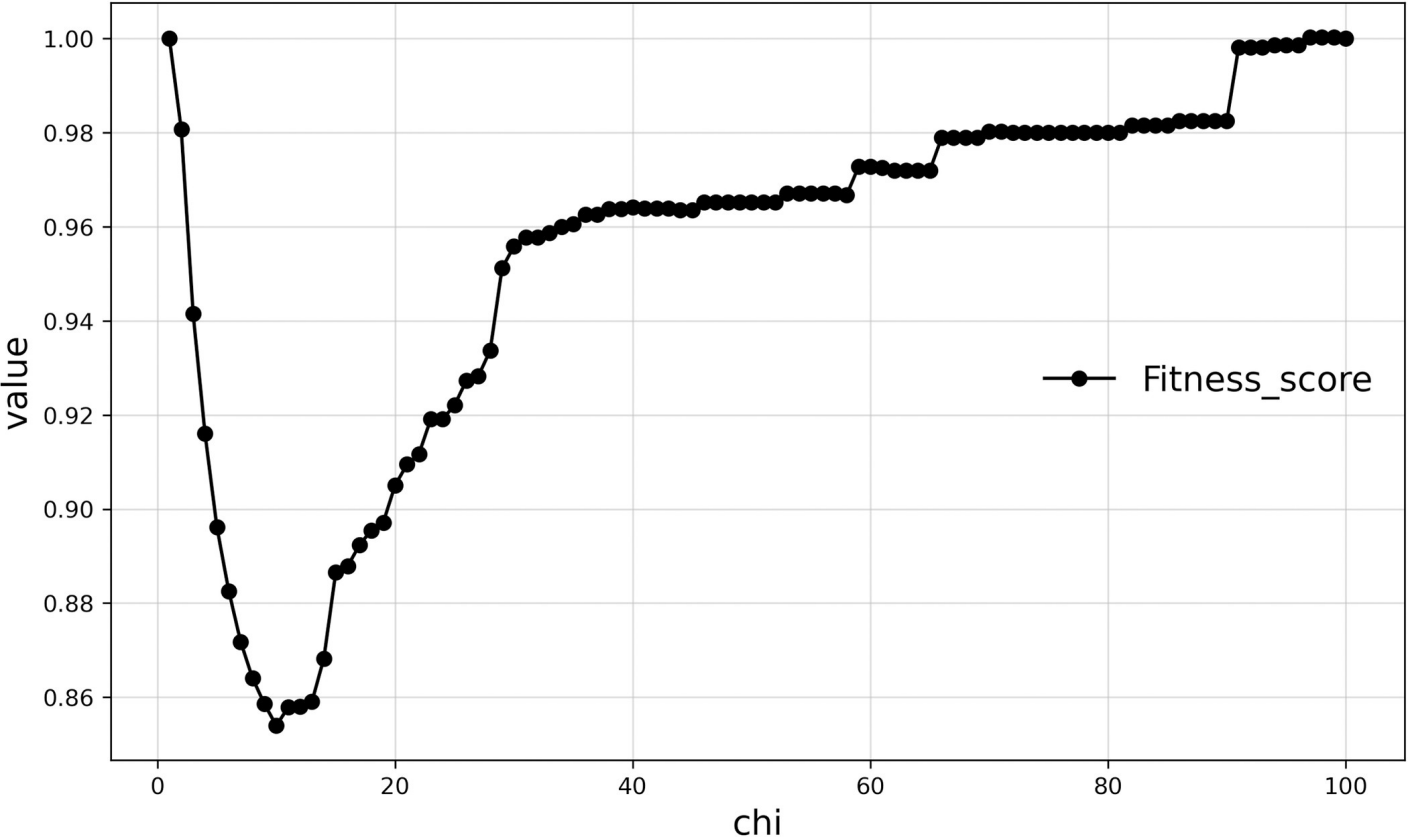
Curve of the fitness function of Haishangqiao village.

**Fig 11:**
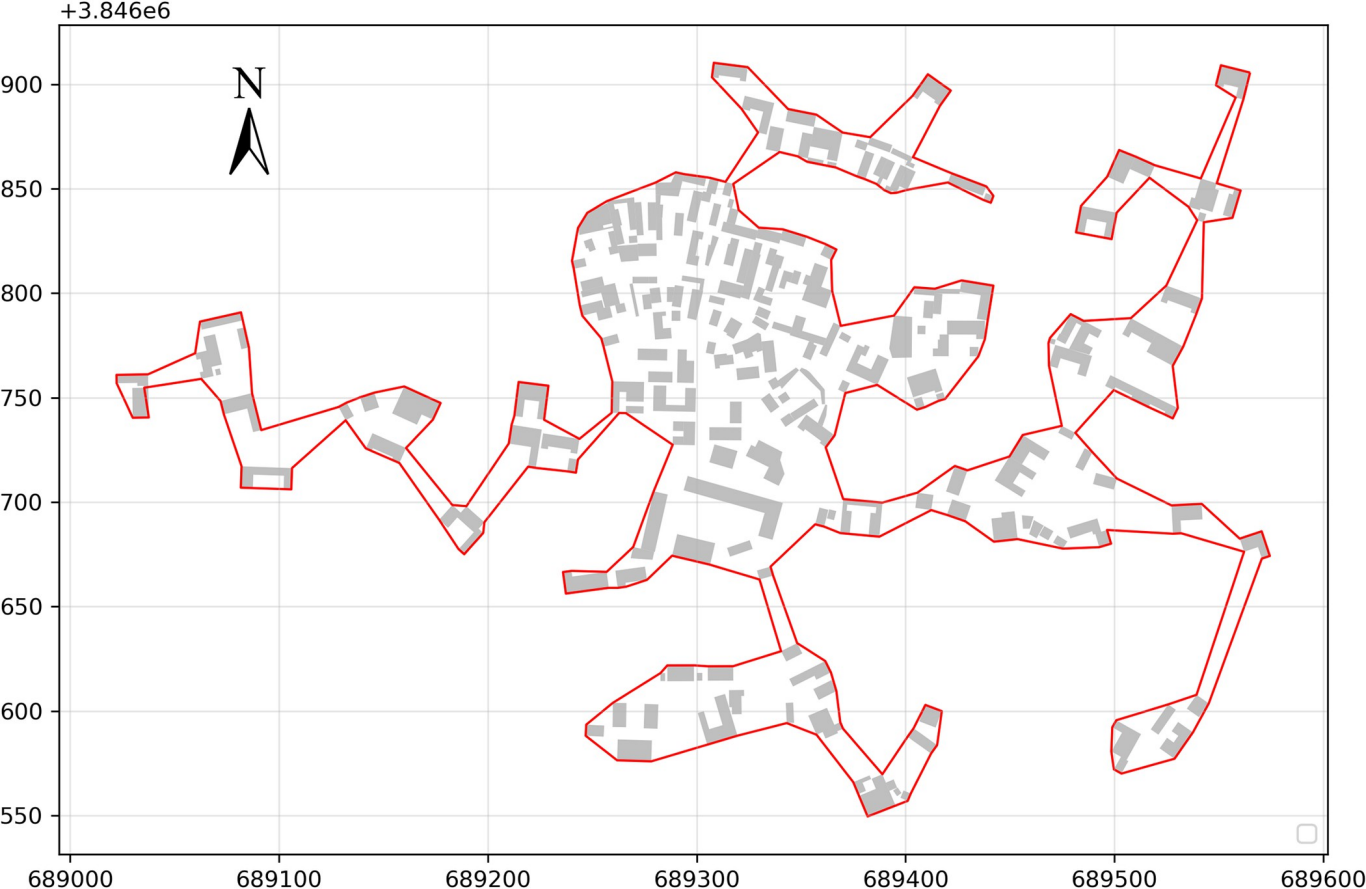
The final boundary of Haishangqiao village (when chi = 9). (The building outline data in Fig 11 was derived from Landsat Science (http://landsat.visibleearth.nasa.gov). This building outline is similar but not identical to the original image).

Village boundaries have to ensure both ease of use at the boundary and customary use of space. They also have to simultaneously satisfy the minimum complexity of the village boundaries and the minimum emptiness. We used the fitness function to find the indicator that balances the two functions. (Fig 10) shows the value of the fitness function in relation to the chi value for Haishangqiao village. We can see that the value of the fitness function decreases rapidly with increasing chi values, then increases rapidly again, and finally increases slowly to reach a maximum value. When chi = 9, the value of the fitness function is minimized and the corresponding village boundaries reach equilibrium in terms of emptiness and complexity. At this point, the length of the virtual line segment is 26.60 m, the emptiness is 0.04, and the complexity is 0.82. The boundary shown in (Fig 11) is the optimal boundary for Haishangqiao village.

## Discussion

### Factors affecting the delineation of traditional village boundaries

The virtual line segments connecting the buildings on the outside of a village are the main factor influencing the delineation of traditional village boundaries. Village boundaries are made up of the walls of the buildings on the outside of a village and the virtual line segments connecting the gaps between the buildings. The walls of the buildings are long-standing, cannot be altered, and are part of the defined solid line segments of village boundaries. The line segments connecting the building gaps are virtual and indeterminate and are the virtual line segments of village boundaries. The fact that virtual line segments can be connected in a variety of ways creates a great deal of uncertainty in boundary delineation. (Fig 3) shows Haishangqiao village’s boundary patterns generated for six lengths of virtual line segments of 3.97, 29.43, 86.01, 142.58, 227.45, and 284.03 m, respectively. The different lengths of virtual line segments form different village boundaries. To reduce the uncertainty of the virtual line segments, we traversed the lengths of 100 virtual line segments as parameters to generate a series of village boundaries.

### Criteria for assessing the reasonableness of traditional village boundaries

The accessibility of village activities and the customary use of village space are the criteria for assessing the reasonableness of traditional village boundaries. The purpose of delineating traditional village boundaries is to clarify the scope of village use. Villagers’, as the main body of village use, reasonable scope of use of a village can be considered village boundaries. The boundary for indoor activities is the boundary of buildings and is part of the physical part of village boundaries. The boundaries of outdoor activities are the gaps between buildings and are the virtual part of village boundaries. Outdoor activities have two types of use: Traffic and stay. Traffic space requires a high level of accessibility; accessibility requires that line segments are as long as possible with as few folding points as possible. Complexity is precisely the indicator that quantifies the ease of access, as evidenced in (Fig 8). Staying space varies from village to village, and the customary use of space by villagers varies due to the natural and human environment in which the village is located. Therefore, the area of customary space should be used as a threshold, and the space in the village should be compared to the threshold; the closer the space is to the threshold, the more it conforms to the villagers’ customs. Meanwhile, the smaller the value, the closer it is to the threshold value and the more the space conforms to the customs of the villagers.

### Factors affecting the complexity of traditional village boundaries

**Fig 12:**
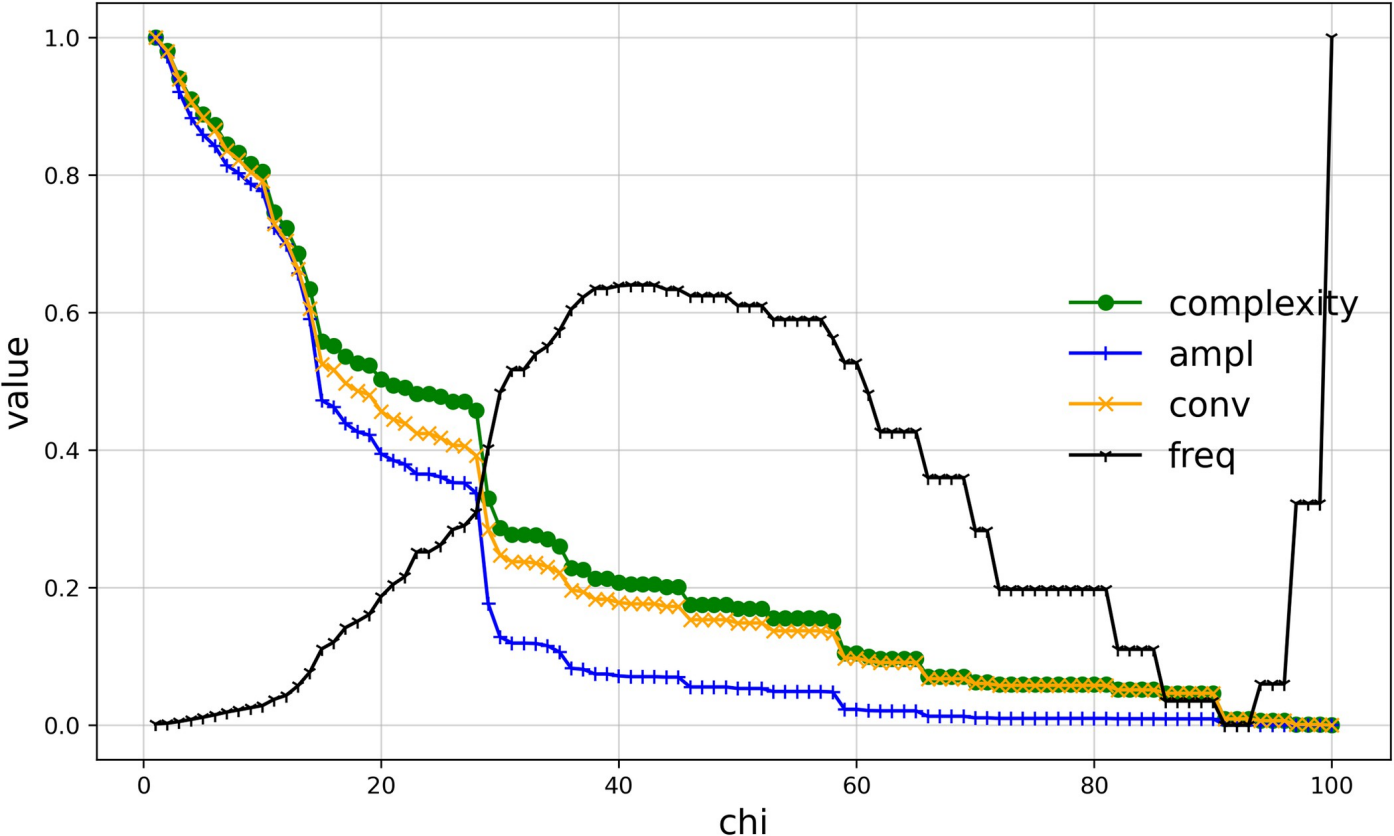
Curve of the relationship among the vibration amplitude, global deviation, frequency, and complexity.

The amplitude of the vibration and global deviation of traditional village boundaries are the main influences on complexity. The complexity of traditional village boundaries is a comprehensive quantitative criterion to assess the ease of use of the space at the edge of a village. The complexity is described by the three parameters of vibration amplitude, global deviation, and vibration frequency of village boundaries. As (Fig 12) shows, the vibration amplitude and global deviation curves of Haishangqiao village’s boundaries are generally consistent with the complexity curve, indicating that these two parameters play a dominant role in complexity. It is clear that the formation of excessively deep depressions between buildings is not conducive to the formation of outdoor enclosed spaces, nor does it provide ease of access for traffic behavior. Again, the depth of the depression formed by the virtual line segment connecting two buildings has a strong influence on the complexity.

### The relationship between emptiness and complexity

**Fig 13:**
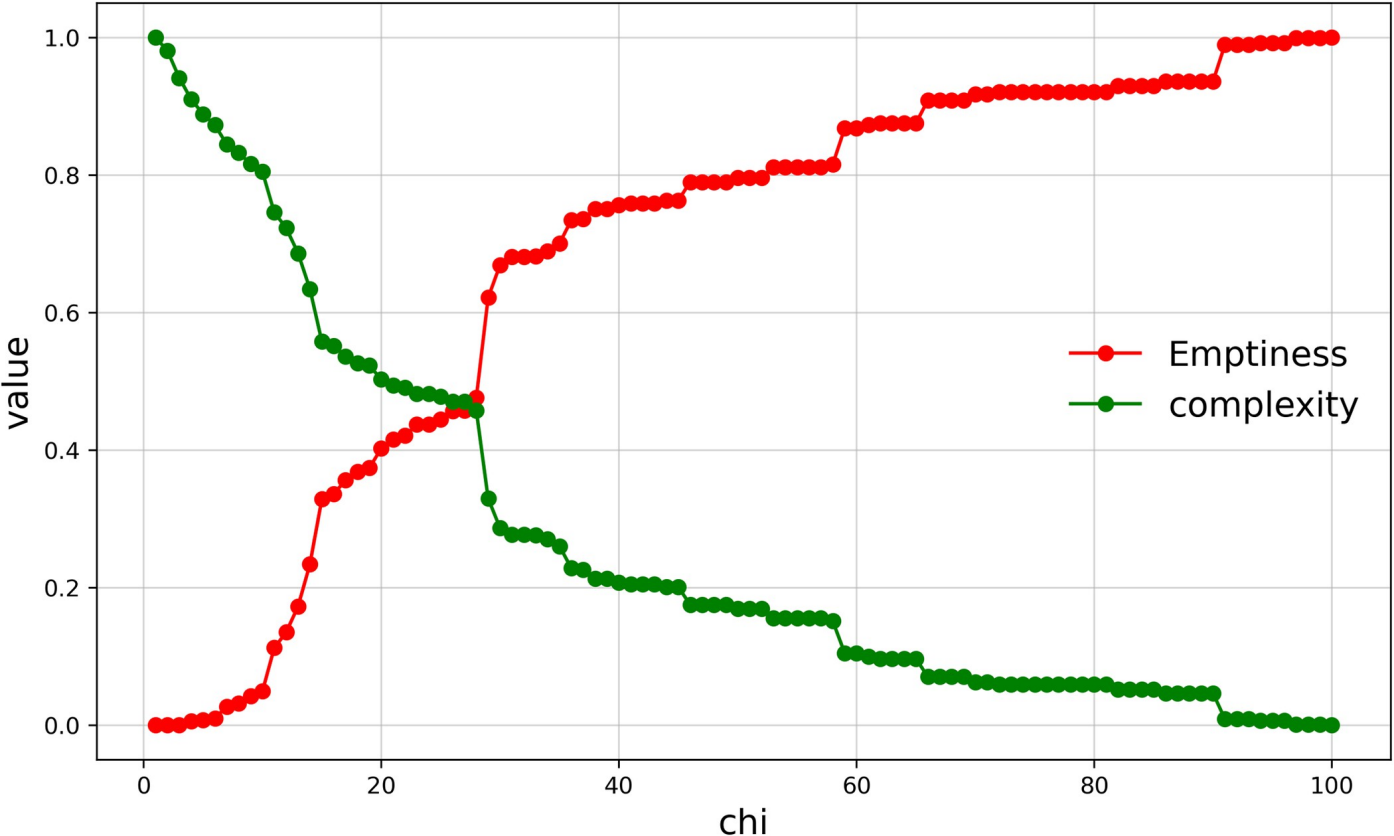
Curve of the relationship between emptiness and complexity.

The emptiness of traditional village boundaries is negatively correlated with complexity. The emptiness is the degree of emptiness of the space within the village boundaries and is an indicator describing the area attributes of a polygon; the complexity is the degree of complexity of the village boundary curve and is an indicator describing the line segments of said polygon. As discussed above, solid and virtual boundaries form the complete boundary of a village, and the variation in the virtual boundary is the main factor influencing the delineation of village boundaries. Different virtual boundaries form village boundaries with different levels of emptiness and complexity. A reasonable village boundary should be one with low emptiness and low complexity. We can clearly see from (Fig 13) that the complexity decreases while the emptiness increases. This is because, as the length of the virtual line segment of the village boundary increases, the distance between the two endpoints that make up the virtual line segment increases, and the number of building entities crossing at once increases, resulting in fewer notches and fewer depressions in the boundary, causing a reduction in the amplitude of vibration and overall deviation of the village boundary, which ultimately leads to a reduction in complexity. Precisely because the boundary notches are reduced and depressions are reduced, the area of space within the boundary is increased, causing an increase in the area of cavity space and ultimately an increase in the emptiness. Therefore, the emptiness is negatively correlated with the complexity.

### Highly practical research

This study provided two highly practical and applied results for rural planning and related research. First, the research results provide a quantitative approach to the delineation of village boundaries in rural planning as a complement to existing methods of boundary delineation. Each model in the study can be used as an influencing factor in the delineation of boundaries in rural planning. Second, all of the models in this study were developed in a computer programming language, and later on, we packaged them into a package on GitHub for download by village planners, builders, researchers, and managers. The user only needs to provide the shp file of the village buildings to automatically complete all of the operations in the text and to generate the optimal village boundaries, or by adjusting the chi parameter values, a series of specific boundaries can be generated for research purposes.

### Limitations and potential improvements

The main limitation of this study is the selection criteria for the threshold θ for calculating the emptiness of a village boundary. The authors of the algorithm, Akdag et al. [45], originally selected a threshold of 1.25 times the mean value of all triangles in the Delaunay triangle network. We applied thresholds of 1.25, 1.5, and 1.75 times the mean triangle value and found that although the fitness score changed, the order of village boundaries did not change, so the threshold θ did not affect the selection of the optimal boundary.

## Conclusions

This paper took the traditional village of Haishangqiao in the Yiluo River Basin as the research object, and generated traditional village boundaries from the solid line segments of traditional village boundaries and the virtual line segments of the connecting lines between buildings. From the perspective of villagers’ use, the evaluation system of traditional village boundary was constructed with convenience and habit as the criteria.

The main findings of this study are as follows. The virtual line segments of traditional village boundaries are the main influencing factors in the delineation of village boundaries, with the length of the virtual line segments influencing the number of buildings crossed at a time, in turn influencing the shape of the boundary curve. The evaluation system of traditional village boundaries includes two main indicators: complexity and emptiness, among which, complexity consists of three sub-indicator systems of vibration frequency, vibration amplitude, and overall deviation. The vibration amplitude and the global deviation of village boundaries play a major role in influencing the complexity of these boundaries. The convenience of villagers’ activities is negatively correlated with the complexity of boundaries, i.e., the more complex a boundary, the less convenient villagers’ activities are. There is also a negative correlation between the emptiness of space within a village and the complexity of village boundaries. If the space inside a village is too empty, this results in a less complex village boundary and use of space of villagers that is not in line with their habits. The optimal village boundary was obtained by finding the optimal values of emptiness and complexity through the adaptation function.

## Supporting information

S1 File (GEOJSON)

S2 File (CSV)
